# 

**Published:** 1906-05-05

**Authors:** 


### MEDICAL APPOINTMENTS AND VACANCIES.

### VACANCIES.

Belgrave Hospital for Children, Clapham Road, S.W.—Senior and Junior Resident Medical Officers.

Birkenhead Borough Hospital.—Junior Resident House Surgeon.

Bodmin, Cornwall County Asylum.—Third Assistant Medical Officer.

Burley-in-Wharfedale, Scalebor Park (Private Asylum).—Assistant Medical Officer.

Canterbury, Kent and Canterbury Hospital.—House Physician.

City of London Hospital for Diseases of the Chest, Victoria Park, E.—Resident Medical Officer. Also Physician to Out-patients. Also Surgeon Dentist.

Colchester, Essex and Colchester General Hospital.—House Surgeon.

Coventry and Warwickshire Hospital.—Junior House Surgeon.

Devonport, Royal Albert Hospital.—Resident Medical Officer.

Fareham, Hants County Asylum.—Second Assistant Medical Officer.

Gosforth, Newcastle-on-Tyne City Asylum.—Assistant Medical Officer.

Kensington General Hospital, late "Queen's Jubilee," Earl's Court, London, S.W.—Assistant Dental Surgeon, Medical Registrar, Gynæcological Registrar (all honorary).

Metropolitan Asylums Board Fever and Small-pox Hospitals.—Assistant Medical Officers.

Metropolitan Asylums Board, Downs School, Sutton, Surrey (for Children with Ringworm).—Assistant Medical Officer.

Miller Hospital and Royal Kent Dispensary, Greenwich Road, S.E.—Junior Resident Medical Officer.

Plymouth, South Devon and East Cornwall Hospital.—Assistant House Surgeon.

Royal Dental Hospital of London, Leicester Square.—Assistant Dental Surgeon.

Sheffield Royal Hospital.—Honorary Assistant Surgeon. Also Two Honorary Assistant Physicians.

Society of Apothecaries of London.—Examiner in Midwifery.

Victoria Hospital for Children, Tite Street, Chelsea, S.W.—Honorary Anæsthetist.

Western General Dispensary, Marylebone Road, N.W.—Honorary Surgeon.

### THE HOSPITAL LIBRARY AND CHARITIES BUREAU.

This institution has been founded as a centre of reference in all matters concerning the establishment and management of charities. It is open to all interested in the subject on payment of a small annual fee, and inquiries by members can be made by post. Write for prospectus to the Librarian, The Hospital Buildings, 28 & 29 Southampton Street, Strand.

Inquiries of the Librarian are answered in this column free of charge. Inquiries to be answered promptly by post must be accompanied by a fee of 2s. 6d

### NOTICE TO CORRESPONDENTS.

All MSS., Letters, Books for Review, and other matters intended for the Editor should be addressed The Editor, "The Hospital," The Hospital Buildings, 28 & 29 Southampton Street, Strand, W.C.

The Editor cannot undertake to return rejected MS., even when accompanied by stamped directed envelope.

**Notice to Advertisers and Subscribers.**

All Advertisements, Orders for Copies of The Hospital, and Business Communications should be addressed to **THE MANAGER (Not to the Editor),** The Hospital, 28 & 29 Southampton Street, Strand London, W.C.

The Cost of Subscription, post free, is as follows:—Per week, 3½d.; per quarter, 3s. 3d.; per half-year, 6s. 6d.; per annum, 13s. Foreign and Colonial:—Per annum, 19s. 6d., payable, in all cases, in advance.

**NOTICE.—Vol. XXXIX. of "The Hospital" (October 1905 to March 1906), in handsome cloth binding, will shortly be on sale at the office of this Journal, price 10s. 6d. Cases for binding the Numbers contained in this Volume 2s. Index to Vol. XXXIX., 3½d., post free.**

**The Monthly Part for May is now ready. Price 1s., by post, 1s. 3d.**

The value of a Book is governed by its contents rather than by the quality of its binding.

Our attention is given almost exclusively to the production of Nursing Text-books, and the fact that our publications are in use in most training schools throughout the English-speaking world is indisputable evidence of their quality.

Therefore, when you are requiring a Nursing Manual, whether as a text-book or work of reference, choose one bearing

**this** 

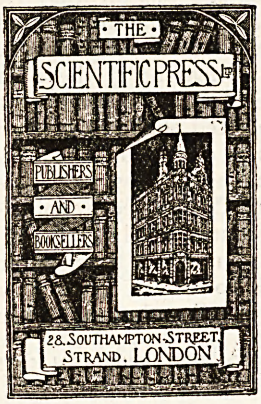

**Sign**

which is a guarantee of quality in all respects. Any work bearing this mark will be found to be the book on its subject.

### These are some of our Books

### HOW TO BECOME A NURSE.

**The Nursing Profession: How and Where to Train.**

Being a Guide to Training for the Calling of a Nurse, with Particulars of Nurse Training Schools in the United Kingdom and Abroad, and an Outline of the Principal Laws affecting Nurses, &c.

Edited by Sir HENRY BURDETT, K.C.B.

Crown 8vo. cloth. **2s.** net; **2s. 4d.** post free.

### HOW TO BECOME A CERTIFIED MIDWIFE.

By E. L. C. APPEL, M.B., B.S., B.Sc.Lond.

Crown 8vo. bound limp cloth. **1s.** net; **1s. 1d.** post free.

### SIMPLE INTRODUCTORY LESSONS IN MIDWIFERY.

By M. LOANE. **1s.** net; **1s. 1d.** post free.

### NOTES ON PHARMACY AND DISPENSING FOR NURSES.

New and Revised Edition.

By C. J. S. THOMPSON. **1s.** post free.

### MENTAL NURSING.

By WILLIAM HARDING, M.D.Ed., M.R.C.P.Lond.

New and Popular Edition. **1s.** post free.

### ART OF MASSAGE.

By A. CREIGHTON HALE.

Second Edition, profusely Illustrated with over 70 special Cuts. **6s.** post free.

### ELEMENTARY TREATISE ON THE LIGHT TREATMENT FOR NURSES.

By JAMES H. SEQUEIRA, M.D.Lond.

Illustrated. **2s. 6d.** net; **2s. 9d.** post free.

### OUTLINES OF ROUTINE IN DISTRICT NURSING.

By M. LOANE, Superintendent of District Nurses, Portsmouth.

Limp cloth. **1s. 6d.** net; **1s. 9d.** post free.

### PRACTICAL HINTS ON DISTRICT NURSING.

By Miss AMY HUGHES. **1s.** post free.

### FEVERS AND INFECTIOUS DISEASES.

**Their Nursing and Practical Management.**

By WILLIAM HARDING, M.D.Ed., M.R.C.P.Lond., Author of "Mental Nursing," &c. **1s.** post free.

### NURSING OF TROPICAL FEVERS.

By S. F. POLLARD, late Sister, Army Nursing Reserve.

**1s. 6d.** net; **1s. 8d.** post free.

The Work is based upon the Personal Experience of the Author abroad.

*We shall be pleased to send you free of cost on receipt of post-card a copy of our Catalogue, which contains many other works on various Nursing subjects equally as good as the above books.*

**The Scientific Press, Ltd.,**

***Catalogue of Nursing Manuals sent post free on application.***

**Publishers of Nursing Text-Books, Charts, and Case-Books of all Descriptions.**

**28 & 29 SOUTHAMPTON STREET, STRAND, LONDON, W.C.**

